# Reproductive Toxicity of Zearalenone and Its Molecular Mechanisms: A Review

**DOI:** 10.3390/molecules30030505

**Published:** 2025-01-23

**Authors:** Qiongxia Lv, Wenjing Xu, Fan Yang, Wenjuan Wei, Xiaoguang Chen, Ziqiang Zhang, Yumei Liu

**Affiliations:** College of Animal Science and Technology, Henan University of Science and Technology, No. 263, Kaiyuan Avenue, Luoyang 471023, China; xwenjing0223@163.com (W.X.); wwj3456453421@163.com (W.W.);

**Keywords:** zearalenone, mycotoxins, toxicity mechanisms, reproductive performance

## Abstract

Zearalenone (ZEA) is one of the common mycotoxins in feeds. ZEA and its metabolites have estrogen-like activity and can competitively bind to estrogen receptors, causing reproductive dysfunction and damage to reproductive organs. The toxicity mechanism of ZEA mainly inhibits the antioxidant pathway and antioxidant enzyme activity, induces cell cycle arrest and DNA damage, and blocks the process of cellular autophagy to produce toxic effects. In animal husbandry practice, when animals ingest ZEA-contaminated feed, it is likely to lead to abortion in females, abnormal sperm viability in males with inflammatory reactions in various organs, and cancerous changes in the reproductive organs of humans when they ingest contaminated animal products. In this paper, we reviewed in detail how ZEA induces oxidative damage by inducing the generation of reactive oxygen species (ROS) and regulating the expression of genes related to oxidative pathways, induces germ cell apoptosis through the mitochondrial and death receptor pathways, and activates the expression of genes related to autophagy in order to induce cellular autophagy. In addition, the molecular detoxification mechanism of ZEA is also explored in this paper, aiming to provide a new direction and theoretical basis for the development of new ZEA detoxification methods to better reduce the global pollution and harm caused by ZEA.

## 1. Introduction

Cereals are the main ingredient in feed formulations for intensive farms, and about 70% of cereal feeds are contaminated with mycotoxin [1]. More than 400 mycotoxins have been identified, which are mainly produced by *Aspergillus*, *Fusarium*, *Penicillium* and *Streptomyces* spp. [2]. Common mycotoxins are mainly aflatoxin B1, zearalenone, vomitoxin, ochratoxin A and T-2 toxin [3]. Most of these toxins are chemically stable, difficult to degrade during feed processing, and widely present in animal feed [4,5]. When animals ingest mycotoxin-contaminated feed, it can cause acute and chronic toxicosis, immune dysfunction, and serious animal death [6]. It can also be enriched into the human body through the food chain, causing miscarriages and fetal malformations in women and reduced sperm viability in men [7]. Addressing the problem of mycotoxin poisoning is therefore essential to protect human and animal health.

Zearalenone (ZEA) is a non-steroidal estrogenic mycotoxin secreted by *Fusarium graminearum* and other *Fusarium* spp. It is also known as a F-2 toxin and is one of the three major mycotoxins in animal feed [8,9]. It is mainly found in cereals such as maize, barley and oats [10]. Under normal conditions, ZEA is white granular, heat-stable and difficult to degrade during food processing and storage [11]. When humans and animals ingest moldy grains, it causes serious damage to the organism. The current situation regarding ZEA contamination is widespread globally, mainly in the northern temperate zone [12]. Studies have shown that in the United States, zearalenone was detected in 27% of 219 maize samples and in 94% of 35 samples of maize DDGS (dry distillers’ dried grains and their solubles), at levels exceeding the recommended safe levels for animal feed [13]; The study showed that the detection of mycotoxin occurrence in feeds in various provinces of China revealed that the rate of ZEA alone contamination reached 96.9%, with average concentrations ranging from 48.1 to 326.8 μg/kg, and most of them were contaminated by the combination of ZEA-DON-AFB [14]; The results of the survey of major mycotoxin contamination in animal feeds showed that the positive detection and exceedance rates of ZEA in pig rations were 76.14% and 14.93%, respectively, and in chicken rations were 66.67% and 20%, respectively [15]; In addition to cereal feed, ZEA was detected at a maximum value of 325.00 μg/kg in oil-rich fruit and seed (*Coix lacryma*) herbs [16]; ZEA was also detected in the extracts of ginseng and American ginseng root, with the highest level of 11.7 mg/kg in ginseng root extract and 2.6 mg/kg in American ginseng root extract [17]. Studies have shown that ZEA and its metabolites have also been detected in animal products, such as pork, lamb and milk, when animals ingested feed contaminated with high levels of ZEA [18]. Unlike other mycotoxins, ZEA and its metabolites have estrogen-like activity, so they will bind to estrogen receptors and exhibit weak estrogenic activity, disturbing estrogen levels in the body and leading to reproductive system disorders [19]. It causes follicular development disorders and ovulation disorders in female animals, reduces the rate of oestrus and the number of ovulations, and even results in the absence of oestrus in female animals [20]. It can also interfere with the secretion of androgen testosterone (T), leading to spermatogenesis disorders, sperm dysplasia and increased malformation rate [21]. In addition, ZEA is carcinogenic [22], reproductively toxic [23], and cytotoxic [24]. In the human body, ZEA binds to α and β estrogen receptors, disrupts the function of the endocrine system [25], disrupts the normal development of the sex organs, triggers inflammation and hypertrophy of the sex organs, induces apoptosis and necrosis of the germ cells, reduces the vitality of the germ cells, and ultimately reduces the reproductive capacity [26,27,28]. Notably, recent studies have found that ZEA can inhibit the growth of endometriotic lesions by antagonizing estrogen when estrogen is sufficient [27]. ZEA is present in the liver for the longest period of time and induces histopathological changes in the liver with subsequent development of hepatocellular carcinoma [29]. ZEA causes oxidative damage to cells by inhibiting normal intracellular DNA and protein synthesis, leading to cytotoxicity [30]. ZEA also produces hemotoxic effects by interfering with the blood coagulation state and altering blood parameters [31]. It has also been found that ZEA can cause mutations in the P53 gene in animals [32]. P53 is an important tumor suppressor gene, and its mutation is associated with tumor development [33]. Therefore, the mutagenic effect of ZEA on the P53 gene may increase the risk of tumors in animals. In addition, it can have a mutagenic effect on bacterial genes, leading to a decrease in bacterial susceptibility to antibiotics and other antimicrobial drugs, exacerbating bacterial resistance and posing a potential threat to public health and clinical care [31,34]. Therefore, solving the problem of ZEA contamination in cereals has become one of the most important concerns of the food and feed industry.

**Figure 1:**
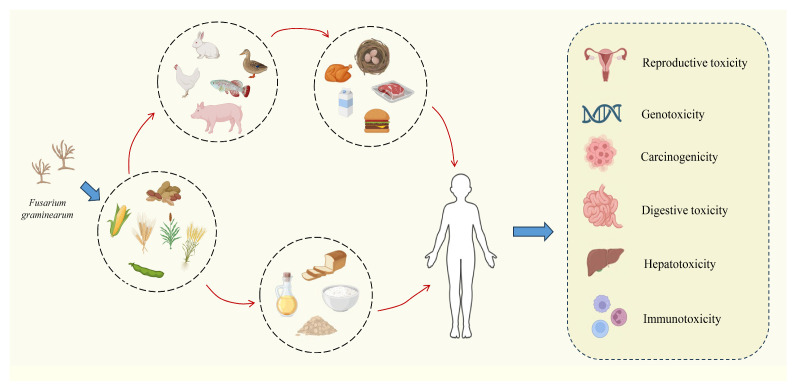
Toxic effects of ZEA.

The main human health effects of ZEA are hormone-dependent diseases such as cervical cancer and prostate cancer [11]. When animals ingest feed contaminated with ZEA, it causes damage to their reproductive organs, causes intestinal dysfunction leading to intestinal inflammation, reduces the body’s immune function, and results in inflammatory reactions in the liver, kidneys, and other organs [23]. ZEA can also cause cytotoxicity due to abnormal cell proliferation and oxidative damage to cells, which can cause serious losses in livestock farming [35]. The main toxic effects caused by ZEA are shown in Figure 1. Therefore, this paper reviewed the current status of global contamination, hazards and toxicity of maize erythromycin and elaborated on the metabolic process, toxic effects and its molecular mechanism of ZEA, with a view to providing a reference for finding drugs or methods to effectively degrade ZEA or mitigate its toxic effects.

## 2. Toxic Effects and Metabolic Processes of ZEA

ZEA is mainly produced by *Fusarium graminearum*, with the molecular formula C_18_H_22_O_5_ and the chemical name 6-(10-hydroxy-6-yloxy-undecadecenyl) β-razololactone [8], the relative molecular mass is 318.364 g/mol. ZEA is a weakly polar compound in the form of white crystals with a melting point of 164–165 °C [36]. It is insoluble in water but soluble in alkaline solutions, benzene, acetonitrile, acetone and alcohol [37]. ZEA is thermally stable and will not be degraded by grinding, extrusion or heating. ZEA is classified as a Group III carcinogen by the International Agency for Research on Cancer [38]. ZEA infests crops mainly during planting, harvesting, transportation and storage of cereals. Studies have shown that ZEA levels in grains are higher in areas with humid air [39].

ZEA and its metabolites have estrogen-like activity that competes with estrogen receptors. ZEA primarily affects the reproductive organs, liver, intestinal tract, and immune system of pigs and ruminants. The hazards of ZEA on different animal organs are shown in Table 1.

**Figure 2:**
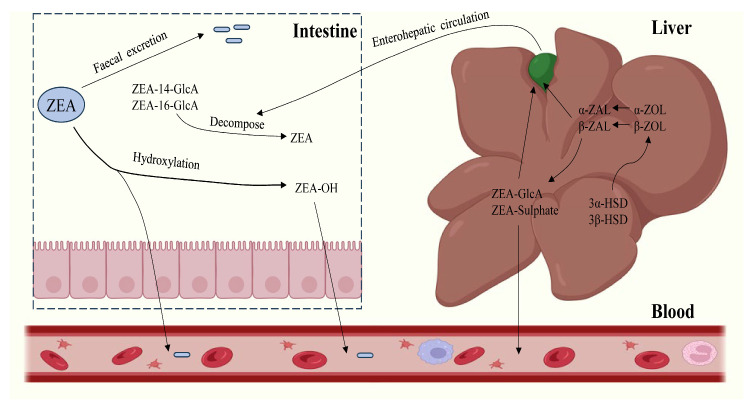
Metabolic processes of ZEA in vivo.

After oral intake of ZEA-contaminated feed, part of it is excreted in urine and feces, while the other part is rapidly absorbed by small intestinal epithelial cells and metabolized in the blood circulation [49]. ZEA is metabolized in the liver mainly by reduction and binding reactions [50]. In addition to the liver, ZEA is also bio-transformed in gastric and intestinal mucosal cells, microorganisms, and the kidneys, where it is metabolized through the following pathways (Figure 2):

ZEA in the presence of 3α/3β-hydroxysteroid dehydrogenases (3α/3β-HSD), ZEA was reduced to 2 isomers of α/β-zearalenol (α/β-ZOL). The α/β-ZOL is structurally similar to ZEA. The difference is that α-ZOL estrogenic activity is nearly 100 times higher than ZEA [51] and is extremely toxic to the body. In contrast, β-ZOL estrogen has low activity and is almost harmless to the organism. In pigs and ruminants, some of the α/β-ZOL can be further reduced to α/β-zearalanol (α/β-ZAL) [52]. ZEA and its metabolites undergo coupling reactions with glucuronic acid (GIcA) and sulfate to form glucuronide chimeras catalyzed by uridine diphosphate-glucuronosyltransferase (UDPGT) and sulfotransferase (SULT), respectively. Glucuronide chimeras do not possess estrogenic bioactivity. Thus, ZEA coupled to GIcA results in a detoxification reaction [53]. Most of the glucuronide chimeras produced are absorbed into the portal vein system via the epithelial cells of the intestinal mucosa and are metabolized in the liver before being transported via the blood circulation to the target organs of the body [54,55]. The remaining glucuronide chimeras are excreted from the body by the bile through the hepatic and intestinal cycles via the feces and urine [56].

## 3. Effects of ZEA Animal Reproductive Organs

### 3.1. Effect of ZEA on the Function of Animal Reproductive Organs

ZEA, as an estrogen-like mycotoxin, competitively binds to the estrogen receptor (ER), causing changes in the spatial structure of the ER, interfering with the expression of downstream target genes, and ultimately impairing the reproductive health of animals [31]. Pigs and ruminants are the most sensitive to the toxic effects of ZEA, and in sows, vulvar swelling and redness, uterine enlargement, mammary gland enlargement, and the presence of ovarian cyst formation were observed after ingestion of ZEA-containing feed [57,58]. Pregnant sows are prone to abortion or premature labor, and the rate of deformities and weak fetuses in newborn piglets increases, even giving birth to dry carcasses and stillbirths [59]. In dairy cows, intake of ZEA-containing feeds has been associated with vulvar swelling, disruption of the estrous cycle, infertility, uterine and mammary gland inflammation, abortion, retained placenta, and vaginitis [60]. Altered oocyte and follicle development in neonatal female mice receiving oral ZEA [61]. Due to the estrogenic effect of ZEA, a low dose (1.5 mg/kg) of ZEA can promote follicular proliferation through the SIRT 1/PGC-1α signaling pathway [62]. It was found that long-term oral administration of low doses of ZEA (20 μg/kg bw and 40 μg/kg bw) to reserve sows induced an increase in estrogens, which caused necrosis of follicular granulosa cells and reduced the proliferative capacity of granulosa cells in the follicle and of connective tissue in the ovarian stroma, especially at the lower doses of ZEA [63]. Long-term oral administration of low doses of ZEA (50 μg/kg bw and 75 μg/kg bw) to bitches enhanced follicular cell apoptosis and reduced follicular cell proliferation, leading to mitochondrial degeneration and nuclear vacuolization in prepubertal bitches [64]. ZEA causes premature puberty in humans [65]. When pregnant women consume foods chronically exposed to ZEA, it can lead to reduced fetal survival, weight loss, and reduced milk production.

ZEA can be toxic to the reproductive system of animals by inducing abnormalities in the testes, seminiferous tubule organization, decreased sperm viability, germ cell apoptosis, and decreased levels of testosterone secretion, among other modes of action [66]. ZEA increased the proportion of immature spermatozoa, decreased the proportion of motile spermatozoa, disorganized the testis, disorganized the arrangement of cells in the seminiferous tubules, decreased the number of layers of spermatogonial cells, and separated spermatogonial cells with vacuolated changes in the epididymis of mice [67]. In contrast, in boars, ZEA often leads to testicular atrophy and reduced sperm concentration [68]. In men, ZEA reduces sperm count and motility [69] and obstruction of sperm production [70]. Feeding a diet containing 40 mg/kg ZEA resulted in decreased libido scores, decreased average daily weight gain, decreased T concentrations, and decreased plasma luteinizing hormone (LH) in boars [71]. It was found that 57.5 μmol/L ZEA induced apoptosis and oxidative stress and decreased the antioxidant capacity of porcine testis cells [72]. Moreover, ZEA significantly reduced testicular interstitial cell viability, significantly reduced the number of live spermatozoa, significantly promoted the mRNA and protein expression levels of the pro-apoptotic gene cysteine aspartate protease 3 (Caspase3), and significantly reduced the mRNA and protein expression levels of the anti-apoptotic gene Bcl-2 [73]. Thus, ZEA can enter the male animal’s organism directly through the feed and damage its reproductive system.

In addition, it has now been shown that mycotoxins can enter the organism through the food chain and cause alterations in epigenetic mechanisms [74]. These alterations may involve mechanisms such as DNA methylation and changes in chromatin conformation, thereby affecting gene expression and the transmission of genetic information [75]. It has been associated with human disease and mycotoxin-induced toxicity in animals, including carcinogenic effects, genotoxicity and reproductive disorders [76,77]. Abnormal DNA methylation due to ZEA exposure has been found to reduce the developmental competence of oocytes and embryos in a variety of animals, including pigs and mice [78,79]. However, there is relatively limited research on how ZEA causes epigenetic changes. Future studies could further explore the effects of ZEA on gene expression and how these effects are transmitted to offspring through epigenetic mechanisms.

### 3.2. Molecular Mechanism of ZEA-Induced Reproductive Organ Damage in Animals

#### 3.2.1. ZEA Produces Excessive ROS and Reduces Antioxidant Enzyme Activities

Oxidative stress refers to the pathological process caused by the imbalance between oxidation and antioxidant mechanisms within the organism [80]. It is an important molecular mechanism that leads to cellular damage [81]. Numerous studies have demonstrated that oxidative stress plays a vital role in the process of ZEA-induced cellular DNA damage [82]. Oxidative stress is the main toxic mechanism through which ZEA causes reproductive damage [83]. High concentrations of ZEA can induce cell apoptosis and oxidative stress, as well as disrupt the structures of the endoplasmic reticulum and mitochondria [84]. In recent years, researchers have gradually shifted their focus from the estrogen signaling pathway to the oxidative stress response in studying the mechanism of ZEA [85]. Oxidative stress refers to the imbalance between oxidation and the antioxidant system in cells and tissues, which is the result of excessive production of oxidative free radicals and related ROS [86]. ROS are mainly formed by mitochondria during the transition from state III respiration to state IV respiration [87]. Generally, ROS serves as the primary mediator for phagocytes to carry out phagocytosis and cell death [88]. However, the imbalance between ROS production and clearance leads to pathological changes in cells [89]. Under certain conditions, such as when consuming feed contaminated with mycotoxins like ZEA, the concentration of free radicals in the body becomes excessively high, disrupting the balance and triggering a cascade reaction of free radicals, leading to oxidative stress and even oxidative damage [90]. ZEA and its metabolites can stimulate the leakage of electrons from the respiratory chain, leading to an increase in ROS [91]. When ROS accumulates to a certain concentration, it can cause damage to mitochondrial DNA, protein denaturation, and lipid peroxidation, resulting in mitochondrial dysfunction [83,84]. This further increases ROS production, leading to oxidative stress and damage [90]. The level of malondialdehyde (MDA) increases significantly, while the activities of total superoxide dismutase (T-SOD), glutathione peroxidase (GSH-Px), and other related antioxidant enzymes decrease significantly [92,93]. The study showed that ZEA treatment of Leydig cells for 24 h resulted in a significant increase in intracellular ROS content and lipid peroxides MDA expression level and a significant decrease in catalase activity, suggesting that ZEA can cause oxidative damage to cells [94]. ZEA was added to the feed, and the oocytes were stained with DCFH-DA fluorescent dye. It was observed that the intensity of ROS fluorescence was significantly enhanced after 46 h of incubation, indicating that the addition of ZEA to the feed induced oxidative stress in porcine oocytes [63].

#### 3.2.2. ZEA Affects the Expression of Oxidative Stress-Related Genes in Germ Cell

Nuclear factor erythroid 2-related factor 2 (Nrf2) is a key factor in cellular regulation of oxidative stress [95]. It mainly functions by eliminating excessive ROS within cells to counteract oxidative stress damage. Nrf2 is particularly sensitive to ROS and plays a vital role in the response to oxidative stress [96]. Nrf2 can participate in regulating various physiological functions within cells, including maintaining the balance of oxidation and antioxidant systems, as well as cell proliferation and metabolism [97]. The target genes of Nrf2 are genes encoding proteins and enzymes involved in oxidative stress detoxification, including HO-1, SOD, CAT, GSH, etc. Activation of the Nrf2 signal can induce the expression of various antioxidant genes, mitigate oxidative damage, and protect cells from the invasion of foreign toxins [98]. Research has reported that ZEA can upregulate the expression levels of genes related to the Nrf2 signaling pathway [99]. Nrf2, by binding to the antioxidant response elements (ARE) in the promoter region, alters the gene expression profile and regulates the expression of downstream antioxidant genes [94]. The Nrf2/ARE pathway is one of the most important defense systems against oxidative damage in the body [86]. The Nrf2 protein activates downstream antioxidant genes to maintain organ homeostasis, relative stability, and oxidative-antioxidative balance by clearing excessive ROS from cells [100]. The activation of Nrf2 is regulated by Kelch-like ECH-associated protein 1 (Keap-1). The Keap-1 protein exists in the cytoplasm of cells and can inhibit Nrf2 when combined with it [101]. When the level of ROS increases in the body, Keap-1 and Nrf2 dissociate, and Nrf2 is then released to exert its antioxidant activity [102]. When animals ingest feed contaminated with ZEA, the expression of Keap-1 protein in cells increases, activating the Nrf2 pathway is suppressed, leading to ROS accumulation and oxidative damage to reproductive organs [103].

**Figure 3:**
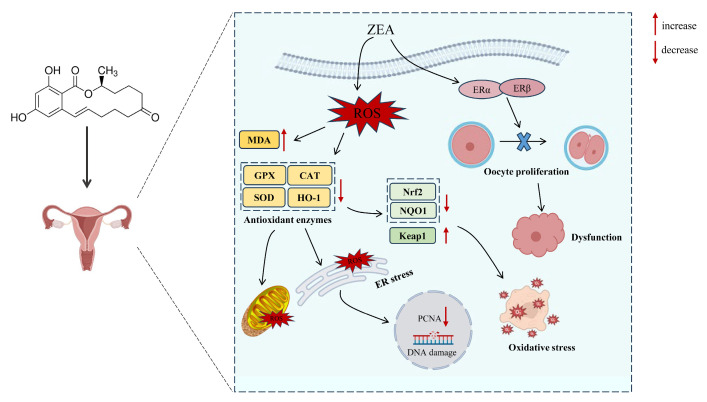
The mechanism of ZEA-induced oxidative damage to reproductive organs.

Oxidative stress plays an important role in the damage to animal reproductive organs caused by ZEA. The main molecular mechanisms are shown in Figure 3. However, the current molecular mechanism of oxidative stress mainly focuses on the Nrf2/Keap1 signaling pathway, and the detailed molecular mechanism of ZEA-induced oxidative stress can be explored in future studies by gene knockdown or the addition of inhibitors and activators. Actively eliminating ROS and increasing the expression and activity of antioxidant enzymes are important measures to prevent and control the damage to animal reproductive cells caused by ZEA. Therefore, in clinical practice, some antioxidants can be added to the feed to enhance the antioxidant capacity of animals and counteract the reproductive toxicity of ZEA.

#### 3.2.3. ZEA Induces Animal Germ Cells Apoptosis Through Mitochondrial Pathways

Cell apoptosis can be divided into two main stages, namely the initiation phase and the execution phase. The initiation phase refers to the opening or closing of a series of regulatory mechanisms within a cell in response to external stimuli [104]. The execution phase is believed to be a cascade amplification reaction where Caspases irreversibly restrict the hydrolysis of their substrates [105]. ZEA-induced cell apoptosis is mainly achieved through the mitochondrial pathway, endoplasmic reticulum pathway, and death receptor pathway [106]. Based on different activation pathways, cell apoptosis can be divided into exogenous pathway and endogenous pathway [107]. The exogenous pathway includes the death receptor pathway, while the endogenous pathway includes the mitochondrial and endoplasmic reticulum pathways [108]. Studies have shown that ZEA primarily induces cell apoptosis through the mitochondrial pathway by generating high levels of ROS [109]. This article mainly introduces how ZEA induces apoptosis in animal reproductive cells through the mitochondrial pathway and death receptor pathway.

The induction of cell apoptosis through the mitochondrial pathway is associated with mitochondrial membrane potential and the expression levels of apoptosis-related proteins [110]. The decline in mitochondrial membrane potential is a hallmark event of early-stage cell apoptosis, indicating the irreversibility of the apoptotic process [111]. ZEA can reduce the mitochondrial membrane potential in reproductive cells, such as Sertoli cells in the testes [112] and granulosa cells in the ovaries [113]. On the other hand, the mitochondrial apoptotic pathway regulates the permeability of the mitochondrial membrane through the early translocation of anti-apoptotic members (such as Bcl-2 and Bcl-xl) and pro-apoptotic members (such as Bad and Bax) of the Bcl-2 protein family [114]. The mitochondria serve as the regulatory center for cell apoptosis, and Cytochrome C (Cyt C), as a key molecule encoded by nuclear genes, is involved in the apoptotic pathway mediated by its release, which is controlled by members of the Bcl-2 protein family [115]. Studies have found that after ZEA acts on cells, it increases cell membrane permeability and intracellular calcium levels, leading to the accumulation of ROS and causing DNA damage [116]. This results in the formation of oligomeric complexes of Bax/Bak, which insert into the outer mitochondrial membrane pores, leading to changes in mitochondrial osmotic pressure, loss of transmembrane potential, and subsequent release of pro-apoptotic molecules, such as Cyt C, Caspase 3, and Caspase 9 from mitochondria into the cytoplasm. Cyt C then binds with apoptotic protease activating factor 1 (Apaf-1) to form an apoptosome, which activates pro-caspase 9, subsequently activating Caspase 3 and Caspase 7 [117,118]. This triggers a Caspase cascade reaction, leading to the translocation of the pro-apoptotic protein Bax to the nucleus and causing cellular changes such as cell shrinkage, nuclear membrane morphological changes, condensed chromatin along the nuclear membrane, dense cytoplasm, and reduced organelle size [119]. Caspase 3 and Caspase 9 increase in a dose-dependent manner, thus inducing cell apoptosis [120]. Studies have shown that ZEA can induce oxidative stress in the testes of mice, resulting in damage to the electron transport chain, ultimately leading to mitochondrial dysfunction and the release of pro-apoptotic mediators, causing cell apoptosis [121]. ZEA can also induce apoptosis in mouse endometrial stromal cells, significantly upregulating the expression levels of the Caspase family in a dose-dependent manner. ZEA can cause mitochondrial damage and apoptosis in porcine endometrial epithelial cells by regulating the JNK/SAPK signaling pathway [122]. Additionally, ZEA can accelerate the division and proliferation of rat endometrial cells [123]. Furthermore, studies have shown that mycotoxins can induce cell apoptosis through pathways such as reducing mitochondrial membrane potential and disrupting mitochondrial morphology [124].

**Figure 4:**
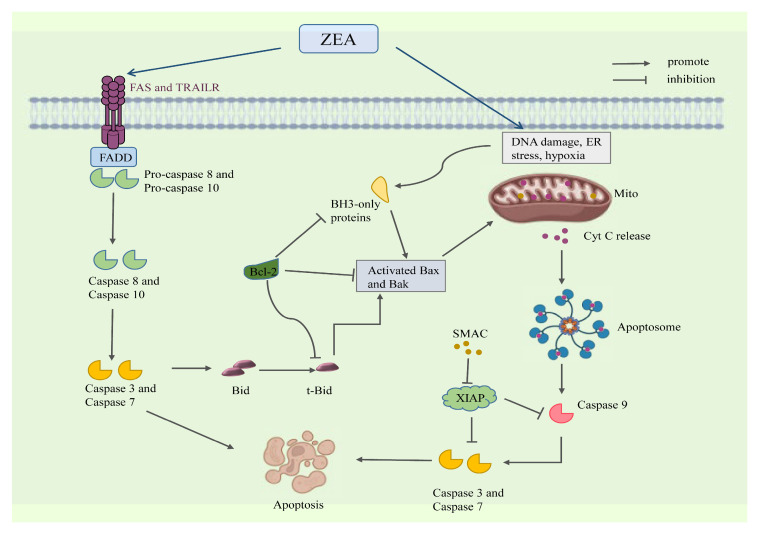
Molecular mechanisms of ZEA-induced apoptosis.

Death receptors are highly expressed in cells, so the induction of cell apoptosis through the death receptor pathway by ZEA is also an important pathway [125]. Known death receptors include Fatty acid synthase (Fas), Tumor necrosis factor receptor 1 (TNFR1), and Death-inducing signaling complex (DISC) [126]. The ligands of death receptors mainly include Fas ligand (FasL) and tumor necrosis factor-alpha (TNF-α) [127]. The Fas pathway primarily induces cell apoptosis by forming trimers consisting of three Fas receptors bound to one FasL, which leads to the clustering of death domains (DDs) [128]. This clustering recruits another protein called Fas-associated death domain (FADD) containing a similar death domain, ultimately resulting in cell apoptosis. FADD can bind to the proform of Caspase 8 [129]. The aggregation of the Fas-FasL complex with FADD and Caspase 8 forms the DISC. The DISC activates the preform of Caspase 8 protein, inducing a cascade of Caspase proteins that ultimately leads to cell apoptosis [130]. In scientific research, the expression of Fas and TNFR 1, as well as the genes associated with DISC formation, such as FADD, TNF receptor-associated death domain (TRADD), and TNF receptor-associated factor 2 (TRAF 2), are mainly detected to verify whether ZEA induces cell apoptosis through the death receptor pathway [131,132]. Death receptors are transmembrane protein receptors belonging to the TNF receptor superfamily. By binding to their ligands, they transmit signals to the Caspase protein family, leading to Caspase activation and subsequently inducing cell apoptosis [130]. Research has found that in rat testicular cells, the expression levels of Fas and FasL genes are positively correlated with ZEA concentration and consistent with protein expression levels. In Sertoli cells of rats treated with ZEA, there is an increase in the ratio of Bax/Bcl-2 and upregulation of FasL, Caspase 3, Caspase 8, and Caspase 9 gene expressions. This suggests that ZEA can activate Caspase 3 to cleave other protein substrates within the cells, leading to cell death through both the mitochondrial pathway and death receptor pathway [133]. Bcl-2 is an anti-apoptotic gene, and its high expression can lead to the accumulation of GSH in the cell nucleus, thereby altering the redox balance within the nucleus and reducing the activity of Caspase, thus inhibiting cell apoptosis [134]. Therefore, the above study suggests that ZEA can induce apoptosis in animal reproductive cells through the death receptor pathway. The specific mechanism of ZEA-induced apoptosis is shown in Figure 4, but the current studies on ZEA on apoptosis are mostly focused on in vitro assays, and the detection of apoptosis-related proteins, and future studies could focus on in vivo assays for validation. And explore the signaling pathway mechanism of apoptosis caused by ZEA, which is crucial for the prevention and control of mycotoxins.

#### 3.2.4. The Relationship Between ZEA and Autophagy of Animal Germ Cells

Autophagy is an adaptive and protective biological process that occurs within cells [135]. Under normal physiological conditions, cells maintain a very low level of autophagy [136]. However, when cells are subjected to various stress conditions such as nutrient deprivation, endoplasmic reticulum stress, and oxidative stress, autophagy is automatically triggered for survival [137]. It is essentially a process of degradation and recycling of cellular components. The microtubule-associated protein light chain 3 (LC3) family is the most important autophagic marker [138]. Detecting the expression of LC3 protein, especially the ratio of LC3-II to LC3-I and the expression of mTOR, can be used to monitor autophagic activity [139]. ZEA has been shown to promote or inhibit cellular autophagy, depending on the dose of ZEA and the target organ of action. Autophagy can be triggered through several interconnected signaling pathways, including the MAPK family [140], PI3K-AKT-mTOR [141], and the ADP/ATP-AMPK [142] signaling pathway.

Research has found that a certain concentration of ZEA can induce autophagy in testicular Sertoli cells through the PI3K/AKT/mTOR signaling pathway [143]. Exposure of rat testicular interstitial cells to ZEA upregulates the expression of LC3-II and Beclin-1, indicating that ZEA can increase the level of autophagy in rat testicular interstitial cells [144]. Exposure to 25, 50, and 100 μmol/L of ZEA can reduce mitochondrial membrane potential in goat testicular Sertoli cells. The phosphorylation levels of Akt and mTOR proteins are significantly decreased, indicating that ZEA can induce autophagy in Sertoli cells through the inhibition of the PI3K/Akt/mTOR signaling pathway [145]. When pigs were fed diets containing 0.15, 1.5 and 3.0 mg/kg ZEA for 32 d, the expression levels of autophagy-related genes LC3, Beclin1, ATG5, ATG7, ATG9, and AMPK, and the anti-apoptosis gene Bcl-2 were significantly increased in the uterus, whereas Bax and mTOR were significantly decreased, leading to hypertrophy of the uterus by activating the AMPK/ mTOR signaling pathway, inducing autophagy and inhibiting apoptosis, leading to uterine hypertrophy [146]. However, it was also found that oral administration of 40 mg/kg bw of ZEA to male Balb/C mice for 5 or 7 consecutive days significantly increased the expression levels of autophagy-related proteins Beclin1, ATG5, P62, and LC3 in testicular tissues and induced cellular autophagy, but autophagic fluxes were blocked, resulting in decreased spermatozoa viability in male mice [147].

**Figure 5:**
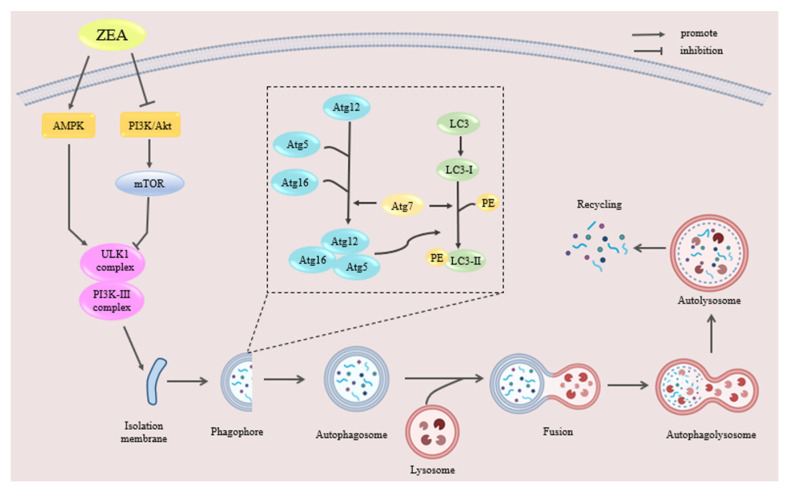
The mechanism of ZEA-induced autophagy in cells.

Moderate autophagy is a normal response of the organism against external stimuli, but excessive cellular autophagy may trigger apoptosis and organ damage. The molecular mechanism of ZEA-induced cellular autophagy is shown in Figure 5. However, the relationship between ZEA-induced apoptosis and cellular autophagy and the detailed mechanism of cellular autophagy is currently unknown. Therefore, we found that ZEA-induced cellular autophagy could be attenuated by targeting the cellular autophagy signaling pathway.

## 4. Conclusions

Domestic and international feeds are widely contaminated with mycotoxins, and ZEA is one of the most contaminated toxins. ZEA has a unique estrogen-like activity that is particularly damaging to the reproductive system of animals. ZEA can cause morphological changes and functional deterioration of reproductive organs in animals, and its toxic effects are still present in their offspring. Currently, it has been found that ZEA mainly exerts its toxic effects on reproductive organs through oxidative stress, cell apoptosis, and autophagy pathways. This paper provides an overview of contaminants in food and feed and their toxicity to humans and animals (pigs, cattle, chickens, mice) worldwide in recent years. This paper also reviews the molecular mechanisms of ZEA-induced reproductive toxicity. There are few reports on natural extracts to alleviate the toxic effects of ZEA, and in the future, we can explore by targeting the Nrf2/HO-1, PI3K/Akt, PINK1/Parkin, MAPK/JNK, and p38/ERK MAPK signaling pathways, to provide targeted strategies for the prevention of ZEA toxicity strategy for the prevention of ZEA toxicity, alleviating cell apoptosis and autophagy, and reducing the damage caused by ZEA to animal reproductive organs. In addition, due to the diversity of ZEA’s toxic effects, the research on the toxic effects and mechanism of ZEA on animal reproductive organs is still incomplete. Therefore, further investigations are required to explore effective strategies for mitigating ZEA’s toxicity and methods for its degradation.

## Figures and Tables

**Table 1:** Summary of the toxic effects of zearalenone on different animal organs.

Cell/Animals	Treatments	Symptomatic	References
Weaned piglet	Weaned piglets were fed 1.5 mg/kg and 3 mg/kg of ZEA for 32 d.	ZEA treatment resulted in massive shedding of epithelial cells from the cecum mucosa, alteration of the number and shape of the cells, and a decrease in the number of cup cells in the cecum as well as in the expression of tight junction proteins.	[40]
Weaned piglet	Weaned piglets were fed 1.04 mg/kg of ZEA for 35 d.	ZEA causes atrophy of ileocecal villi in weaned piglets and reduces the expression of nuclear proliferation antigen (PCNA) in the mucosal epithelium of the duodenum and jejunum, which in turn affects intestinal absorption.	[41]
Gestating sow	Gestating sows were fed diets containing 1, 2, and 10 mg/kg for the 7–14 d of pregnancy, respectively.	Increased thickness of the endometrial epithelial cell layer, the number of erythrocytes in the endometrial capillaries and the number of autophagosomes in the endometrium of gestating sows following the feeding of ZEA-containing diets led to an inflammatory response in the endometrium, which in turn affected the process of implantation of the embryo and the size of the embryo.	[42]
Boars	Boars were fed diets containing 0.8 mg/kg ZEA for 28 d.	ZEA treatment significantly altered the boar's cecum microbiota by increasing the relative abundance of *Lactobacillus* and *Anabaena*.	[43]
Balb/c mice	Mice were given 40 mg/kg bw of ZEA by gavage for 2 d.	ZEA alters immune parameters in mice, leading to pituitary adenomas and nephrotoxicity.	[44]
Balb/c mice	Mice were given 40 mg/kg bw of ZEA by gavage for 5 d.	ZEA elevated the levels of TNF-α and IL-1β in mice liver. Increased phosphorylation of NF-κB p65 and IκBα induced oxidative stress and inflammatory injury in mice by activating the SIRT1/Nrf2 signaling pathway.	[45]
Domestic rabbit	Rabbits were fed diets containing 10, 100 μg/kg for 14 d.	Low concentrations of ZEA (10 μg/kg) significantly increased the activity of alkaline phosphatase (ALP); high concentrations of ZEA (100 μg/kg) significantly increased the activities of ALP, ALT, AST, γ-glutamyltransferase (GGT), and lactate dehydrogenase (LDH), and damaged hepatocytes.	[46]
Chickens	Chicks were given 1.25, 2.5 and 5.0 mg/kg of ZEA by gavage for 7 d.	Diffuse necrosis and focal steatosis of hepatocytes appear in the liver; renal medulla and renal tubules show bruising, glomerular atrophy, swelling and granular degeneration of proximal tubular epithelial cells, etc. AST, ALT activity and the levels of urea (UREA) and uric acid (UA) are significantly elevated, and the levels of total protein (TP) and albumin (ALB) are significantly lowered, which destroys the normal hepatic and renal tissue structures, leading to abnormal liver and kidney functions.	[47]
Cow mammary epithelial cells	Cells were treated with 5, 15 and 30 μM ZEA for 24 h.	Increasing ROS content, decreasing mitochondrial membrane potential, increasing mRNA of endoplasmic reticulum stress markers (GRP78, HSP70, etc.), pro-apoptotic genes Bax and Caspase 3 expression, and decreasing inhibitory gene Bcl2 expression, thus inducing apoptosis of mammary epithelial cells in dairy cows.	[48]

## Data Availability

Not applicable.
